# Chronic asymptomatic pyuria precedes overt urinary tract infection and deterioration of renal function in autosomal dominant polycystic kidney disease

**DOI:** 10.1186/1471-2369-14-1

**Published:** 2013-01-07

**Authors:** Jin Ho Hwang, Hayne Cho Park, Jong Cheol Jeong, Seon ha Baek, Mi Yeun Han, Kitae Bang, Jeong Yeon Cho, Suk Hee Yu, Jaeseok Yang, Kook-Hwan Oh, Young-Hwan Hwang, Curie Ahn

**Affiliations:** 1 Department of Internal Medicine, Seoul National University Hospital, 101 Daehak-no, Jongno-gu, Seoul, 110-744, South Korea; 2 Research Center for Rare Diseases, Seoul National University Hospital, Seoul, South Korea; 3 Department of Internal Medicine, Daejeon Eulji University Hospital, Daejeon, South Korea; 4 Department of Radiology, Seoul National University Hospital, Seoul, South Korea; 5 Department of Internal Medicine, Chung-Ang University Hospital, Seoul, South Korea; 6 Transplantation Center, Seoul National University Hospital, Seoul, South Korea; 7 Department of Internal Medicine, Eulji General Hospital, Seoul, South Korea

**Keywords:** Polycystic kidney disease, Chronic renal failure, Glomerular filtration rate, Pyuria, Urinary tract infection

## Abstract

**Background:**

Urinary tract infection (UTI) occurs in 30%-50% of individuals with autosomal dominant polycystic kidney disease (ADPKD). However, the clinical relevance of asymptomatic pyuria in ADPKD patients remains unknown.

**Methods:**

We retrospectively reviewed medical records of 256 ADPKD patients who registered to the ADPKD clinic at Seoul National University Hospital from Aug 1999 to Aug 2010. We defined the asymptomatic pyuria as more than 5-9 white blood cells in high-power field with no related symptoms or signs of overt UTI. Patients were categorized into 2 groups depending on its duration and frequency: Group A included non-pyuria and transient pyuria patients; Group B included recurrent and persistent pyuria patients. The association between asymptomatic pyuria and both the development of overt UTI and the deterioration of renal function were examined.

**Results:**

With a mean follow-up duration of 65.3 months, 176 (68.8%) out of 256 patients experienced 681 episodes of asymptomatic pyuria and 50 episodes of UTI. The annual incidence of asymptomatic pyuria was 0.492 episodes/patient/year. The patients in group B showed female predominance (58.5% vs. 42.0%, *P*=0.01) and experienced an upper UTI more frequently (hazard ratio: 4.612, 95% confidence interval: 1.735-12.258; *P*=0.002, adjusted for gender and hypertension). The annual change in estimated glomerular filtration rate (ΔeGFR) was significantly larger in magnitude in group B than in group A (-2.7±4.56 vs. -1.17±5.8, respectively; *P*=0.01). Age and Group B found to be the independent variables for ΔeGFR and developing end-stage renal disease (16.0% vs. 4.3%, respectively; *P*=0.001).

**Conclusions:**

Chronic asymptomatic pyuria may increase the risk of developing overt UTI and may contribute to declining renal function in ADPKD.

## Background

Autosomal dominant polycystic kidney disease (ADPKD) is the most prevalent hereditary kidney disease with the incidence of 1 case per 400-1,000 live births [1]. ADPKD is the 4^th^ most common cause of end-stage renal disease (ESRD), occurring in 4%-10% of ESRD patients who initiate renal replacement therapy. However, patients exhibit renal function impairment only when their kidneys are loaded with cysts [1,2].

Urinary tract infection (UTI) is one of the most common renal complications in ADPKD. Approximately 30%-50% of ADPKD patients experience an episode of UTI in their lifetime. UTI is more prevalent in females, and a gram-negative enteric bacterium is the most common pathogen[2-5]. Aside from cystitis, upper UTIs—including renal cyst infection and acute pyelonephritis (APN)—are serious enough to result in long-term hospital care and call for aggressive antibiotic therapy. Despite careful management, UTI often recurs or results in treatment failure [4-6]. Moreover, asymptomatic pyuria is frequently observed in ADPKD patients and often persists or relapses without treatment [7]. However, the clinical implications of asymptomatic pyuria in ADPKD have not been investigated.

Risk factors for kidney failure in ADPKD patients have been suggested to include the *PKD1* gene mutation, hypertension, large kidney size, male gender, proteinuria, and a younger age at diagnosis [8,9]. However, some studies have investigated the impact of UTI on renal function in ADPKD patients [9,10]. In 2006, Ahmed et al. reported that UTI is a risk factor for deteriorating renal function in ADPKD patients along with other traditional risk factors [9]. In another retrospective study, UTI was suggested to be a cause of renal deterioration based on the finding that renal function was preserved better in the group of patients who used prophylactic antibiotics than in the control group [10].

In the present study, we investigated the impact of asymptomatic pyuria on the development of overt UTI and the deterioration of renal function in ADPKD patients.

## Methods

### Study population

This study was performed as a retrospective, single-center, case–control study. Among the patients who registered at the ADPKD clinic at Seoul National University Hospital from Aug 1999 through Aug 2010, we retrospectively reviewed medical records of 311 individuals and collected data from 256 adult patients who received ≥ 2 urinalysis tests during their follow-up period. Patients were seen in the clinic based on their renal function: every 3-6 months for those in CKD stage I-II, every 2-3 months for those in CKD stage III, and every 6-8 weeks for those in CKD stage IV. In addition to the disease severity, we followed up patients more frequently after overt UTI episodes or when they needed acute managements such as high blood pressure or new onset hematuria. Patients with either an estimated glomerular filtration rate (eGFR) of < 15 ml/min/1.73m^2^ or receiving renal replacement therapy were excluded from the analysis (n = 17). Patients with a brief follow-up duration of < 6 months (n = 38) were also excluded from the analysis.

### Definition

ADPKD was diagnosed according to the Unified Criteria for Ultrasonographic Diagnosis of ADPKD as proposed by Pei et al [11]. We defined asymptomatic pyuria as higher than 5-9 white blood cells/high-power field (WBC/HPF) in a random urine sample without symptoms related to overt UTI. The duration of pyuria episode was defined from detection to resolution of pyuria in a subsequent urinalysis.

We categorized the patients into following 4 groups depending on the duration and frequency of the asymptomatic pyuria: no pyuria, transient pyuria, recurrent pyuria, and persistent pyuria. The no pyuria group included patients who did not experience pyuria during the study period. The transient pyuria group included patients who had fewer than 3 episodes of pyuria and patients who had ≥ 3 episodes of pyuria with inter-episode intervals longer than 6 months. Recurrent pyuria was defined as ≥ 3 episodes of pyuria within 6 months. The persistent pyuria group was defined as patients who had pyuria for ≥ 1 month. After this classification, the no pyuria and transient pyuria groups were classified as group A, and the recurrent pyuria and persistent pyuria groups were classified as group B (the chronic pyuria group).

To evaluate the effect of pyuria on the occurrence of subsequent UTI or renal function decline, we additionally divided the patients into two groups based on the number of pyuria episodes in the first year of follow up. Group^no pyuria/1st year^ was defined as the group who did not experience any pyuria episodes, and Group^1-4 pyuria/1st year^ was the sum of patients who experienced 1-4 pyuria episodes during the first year of follow up.

Acute cystitis was diagnosed based on the presence of lower urinary tract symptoms such as dysuria, a burning sensation upon voiding, frequency, urgency, or suprapubic pain with no clinical evidence of an upper urinary tract infection. Upper UTI included both APN and renal cyst infection. The APN was diagnosed clinically when patients develop rapid urinary symptoms with high fever and flank pain and the urinalysis shows signs of urinary tract infection [12]. Cyst infection was confirmed if a cyst aspirate shows definite microorganism or neutrophils debris. In addition, cyst infection was clinically suspected when fever >38.5°C develops without definite pyuria, focal tenderness over the suspected cyst, positive blood culture without urine culture positivity [13]. The imaging tools such as magnetic resonance imaging, ultrasonography, computed tomography, or positron emission tomography were taken to supplement the diagnosis of cyst infection.

Serum creatinine (sCr) was measured using the Jaffe method [14]. The Chronic Kidney Disease Epidemiology (CKD-EPI) equation was used to calculate eGFR [15,16]. The onset of ESRD was defined as the time at which renal replacement therapy (hemodialysis, peritoneal dialysis, or transplantation) was initiated [17].

### Statistical analysis

All statistical analyses were performed using SPSS version 17.0 (SPSS Inc., Chicago, IL, USA). Continuous variables were expressed as the mean ± standard deviation. The independent *t*-test or Mann–Whitney U test was used to compare the continuous variables among the groups, and the Chi-square test was used to analyze the categorical variables. The one-way analysis of variance (ANOVA) was used to compare the continuous variables among the 3 groups. The log rank test was used to compare UTI occurrence between group A and group B, and the multiple Cox regression model was used to identify risk factors. Differences with *P* < 0.05 were considered to be statistically significant.

This study was approved by the Institutional Review Board of Seoul National University Hospital (H-0901-046-269). Informed consent was obtained from the subjects in accordance with the Declaration of Helsinki.

## Results

### Clinical Characteristics Associated with Asymptomatic Pyuria in ADPKD Patients

The baseline characteristics of the 256 patients are presented in Table 1. The age of the study participants was 48.1 ± 12.8 years, and almost the same numbers of male and female subjects were enrolled. The mean follow-up duration was 65.3 ± 43.2 months, and the initial eGFR was 91.1 ± 29.2 ml/min/1.73m^2^. Asymptomatic pyuria was observed in 26.2% of the patients at their first office visit. The incidence of asymptomatic pyuria was 0.492 episodes/patient/year. One hundred and seventy-six patients (68.8%) experienced ≥ 1 episode of asymptomatic pyuria during the course of their follow-up. Asymptomatic pyuria accounted for 93.2% of the total number of infection episodes (Table 2). The patients in group B (n = 94, 36.7%) comprised a higher percentage of females (58.5% vs. 42.0% for group A; *P* = 0.01) and had a longer follow-up duration (88.5 ± 37.8 vs. 51.8 ± 40.4 months for group A; *P* < 0.001). The prevalence of hypertension, diabetes mellitus, and urinary stones did not differ between the groups (Table 1, *P* > 0.05).

**Table 1:** Baseline characteristics of the subjects in this study

		**Group A (n = 162)**	**Group B (n = 94)**	**Total (n = 256)**	***P *value**
Age^*^		48.2 ± 13.7	47.8 ± 11.2	48.1 ± 12.8	NS
Female	number (%)	68 (42.0)	55 (58.5)	123 (48.0)	0.01
Follow-up duration^*^	months	51.8 ± 40.4	88.5 ± 37.8	65.3 ± 43.2	<0.001
Initial eGFR^*†^	ml/min/1.73m^2^	92.4 ± 27.1	88.7 ± 32.47	91.1 ± 29.2	NS
Diabetes mellitus	number (%)	2 (1.2)	1 (1.1)	3 (1.2)	NS
Hypertension	number (%)	99 (61.1)	68 (72.3)	167 (65.2)	NS
Urinary stone	number (%)	24 (14.8)	16 (17.0)	40 (15.6)	NS

Group A included non-pyuria and transient pyuria patients; group B included recurrent and persistent pyuria patients.

*: mean ± standard deviation (SD).

^†^: Estimated glomerular filtration rate (eGFR) was calculated using the CKD-EPI equation (ml/min/1.73m^2^).

eGFR, estimated glomerular filtration rate.

**Table 2:** Incidence of asymptomatic pyuria and overt urinary tract infection

	**Patients (n = 256)**	Episodes (n = 731)*
No pyuria	80 (31.2%)	-
Asymptomatic pyuria	176 (68.8%)	681 (93.2%)
Cystitis	-	17 (2.3%)
Acute pyelonephritis	-	13 (1.8%)
Cyst infection	-	20 (2.7%)

*Some patients experienced ≥ 1 infection episode and/or ≥ 1 type of infection. Asymptomatic pyuria comprises 93.2% of all 731 infection episodes.

Urine culture was performed in only 4.6% of the patients with asymptomatic pyuria. In most of asymptomatic pyuria cases (95.4%), urine culture was not performed. Among those who underwent urine culture study, 6.5% showed negative results. The most common microorganisms were *Escherichia coli* (34.5%), followed by *Klebsiella pneumoniae* (13.8%), *Staphylococcus epidermidis* (11.6%), *Streptococcus viridans* group (9.3%), *Pseudomonas aeruginosa* (7.0%), *Streptococcus agalactiae* (7.0%), *Serratia marcescens* (3.4%), *Corynebacterium species* (3.4%), and *Staphylococcus aureus* (3.4%).

### Association of Asymptomatic Pyuria with Overt UTI

**Figure 1:**
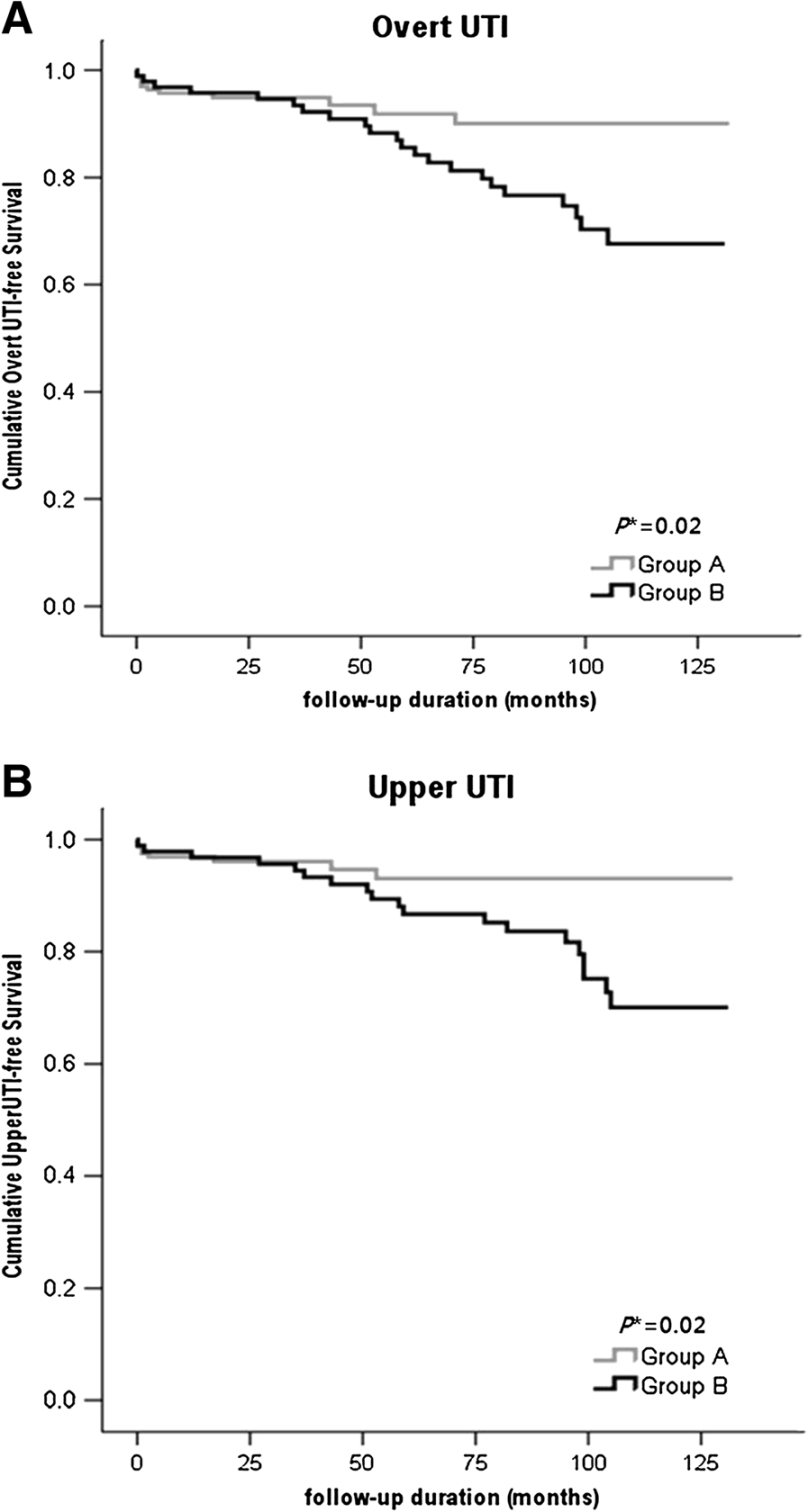
**Kaplan-Meier curves for the occurrence of overt urinary tract infection in group A and group B.A**. The occurrence of overt UTI. Compared to group A, group B had shorter periods of overt UTI-free survival (*P* =0.02). **B**. The occurrence of upper UTI (APN and cyst infection). Compared to group A, group B had shorter periods of upper UTI-free survival (*P* = 0.02).* The comparison between groups was performed using the log rank test. UTI, urinary tract infection.

During the observational period, 33 (12.9%) patients developed an overt UTI. The follow-up duration was longer for the overt UTI group than for the patients without an overt UTI (81.6 months vs. 62.4 months, respectively; *P* = 0.01). More patients in group B experienced an overt UTI during their follow-up than the patients in group A (66.7% vs. 32.3%, respectively; *P* < 0.001; Table 3). Age, gender, diabetes mellitus, hypertension, and urinary stones did not increase the risk of developing an overt UTI. Compared to group A, group B had shorter periods of overt UTI-free survival and shorter periods of upper UTI-free survival (Figure 1). In addition, when we used prospective groups (Group^no pyuria/1st year^ and Group^1-4 pyuria/1st year^) based on the number of pyuria episodes in the first year of follow up, the Group^1-4 pyuria/1st year^ demonstrated the even shorter periods of both overt UTI-free survival and upper UTI-free survival (Additional file 1: Figure S1). A Cox regression analysis was performed to determine whether group B is an independent factor for overt UTI and upper UTI. The forward stepwise method was used, and age, gender, hypertension, urinary stones, group B, and initial eGFR were included in the final analysis. In the univariate analysis, group B and lower initial eGFR were the factors associated with overt UTI. Multiple Cox regression analysis revealed that group B is an independent factor for overt UTI (hazard ratio 4.636, 95% confidence interval 1.898 to 11.323; *P* = 0.001; Table 4). In addition, group B independently increased the risk of upper UTI compared to group A (hazard ratio 4.612, 95% confidence interval 1.735 to 12.258; *P* = 0.002; Table 5). Because the incidence of acute cystitis was low, a Cox regression analysis for acute cystitis was not performed.

**Table 3:** Clinical characteristics according to the occurrence of overt urinary tract infection

		**No UTI (n = 223)**	**Overt UTI (n = 33)**	**Total (n = 256)**	***P *value**
Age^*^		47.6 ± 13.0	51.2 ± 11.8	48.2 ± 12.9	NS
Female	number (%)	102 (45.7)	21 (63.6)	123 (48.0)	NS
F/U duration^*^	Months	62.4 ± 42.7	81.6 ± 39.5	66.2 ± 42.9	0.01
HTN (initial)	number (%)	141 (63.2)	26 (78.8)	167 (65.2)	NS
Stone (initial)	number (%)	34 (15.2)	6 (18.2)	40 (15.6)	NS
Group B	number (%)	72 (32.3)	22 (66.7)	94 (36.7)	<0.001
Initial eGFR^*^†	ml/min/1.73m^2^	92.0 ± 28.9	84.7 ± 30.8	91.1 ± 29.2	NS

*: mean ± standard deviation (SD).

†: Estimated glomerular filtration rate (eGFR) was calculated using the CKD-EPI equation (ml/min/1.73m^2^).

eGFR, estimated glomerular filtration rate; F/U, follow-up; HTN, hypertension.

**Table 4:** Factors associated with the occurrence of overt UTI

	**Univariate**			Multivariate*		
	**HR**	**95% CI**	***P *value**	**HR**	**95% CI**	***P *value**
Age	1.009	0.981-1.037	NS	-	-	-
Female	1.884	0.924-3.839	NS	1.735	0.812-3.707	NS
HTN (initial)	1.849	0.797-4.293	NS	1.865	0.796-4.369	NS
Stone (initial)	1.383	0.570-3.356	NS	-	-	-
Group B	4.717	1.934-11.506	0.001	4.636	1.898-11.323	0.001
Initial eGFR^†^	0.987	0.976-0.998	0.03	0.989	0.978-0.999	0.03

Data were analyzed using the Cox regression, backward stepwise method in the multivariate analysis.

*Adjusted for gender, HTN, pyuria group and initial eGFR.

†: Estimated glomerular filtration rate (eGFR) was calculated using the CKD-EPI equation (ml/min/1.73m^2^).

CI, confidence interval; eGFR, estimated glomerular filtration rate; HR, hazard ratio; HTN, hypertension; UTI, urinary tract infection.

**Table 5:** Factors associated with the occurrence of upper UTI

	**Univariate**			Multivariate*		
	**HR**	**95% CI**	***P *value**	**HR**	**95% CI**	***P *value**
Age	1.004	0.973-1.035	NS	-	-	-
Female	1.502	0.695-3.244	NS	1.268	0.554-2.905	NS
HTN (initial)	1.410	0.593-3.355	NS	1.406	0.585-3.378	NS
Stone (initial)	1.108	0.382-3.208	NS	-	-	-
Group B	4.698	1.770-12.470	0.002	4.612	1.735-12.258	0.002
Initial eGFR†	0.987	0.975-0.999	0.04	0.989	0.977-1.000	0.05

Data were analyzed using the Cox regression, backward stepwise method in the multivariate analysis.

*Adjusted for gender, HTN, pyuria group and initial eGFR.

†: Estimated glomerular filtration rate (eGFR) was calculated using the CKD-EPI equation (ml/min/1.73m^2^).

CI, confidence interval; eGFR, estimated glomerular filtration rate; HR, hazard ratio; HTN, hypertension; UTI, urinary tract infection.

### Clinical Manifestations of Overt UTI in ADPKD

Among 33 patients with an overt UTI, 4 experienced both acute cystitis and an upper UTI, 6 had only acute cystitis and 23 had only upper UTI. The incidence of acute cystitis was 0.012 episodes/patient/year, and the incidence of upper UTI was 0.023 episodes/patient/year. A total of 27 patients experienced 33 episodes of upper UTI (13 episodes of APN and 20 episodes of renal cyst infection). The most common pathogen associated with upper UTI was *E. coli* (24.2%), followed by *K. pneumoniae* (9.1%). One patient had a renal abscess without a cyst infection, and this was categorized as APN. All four patients (14.8%) who experienced repeated upper UTI episodes were in group B.

### Annual Reduction Rate of eGFR in ADPKD Patients with Asymptomatic Pyuria

**Figure 2:**
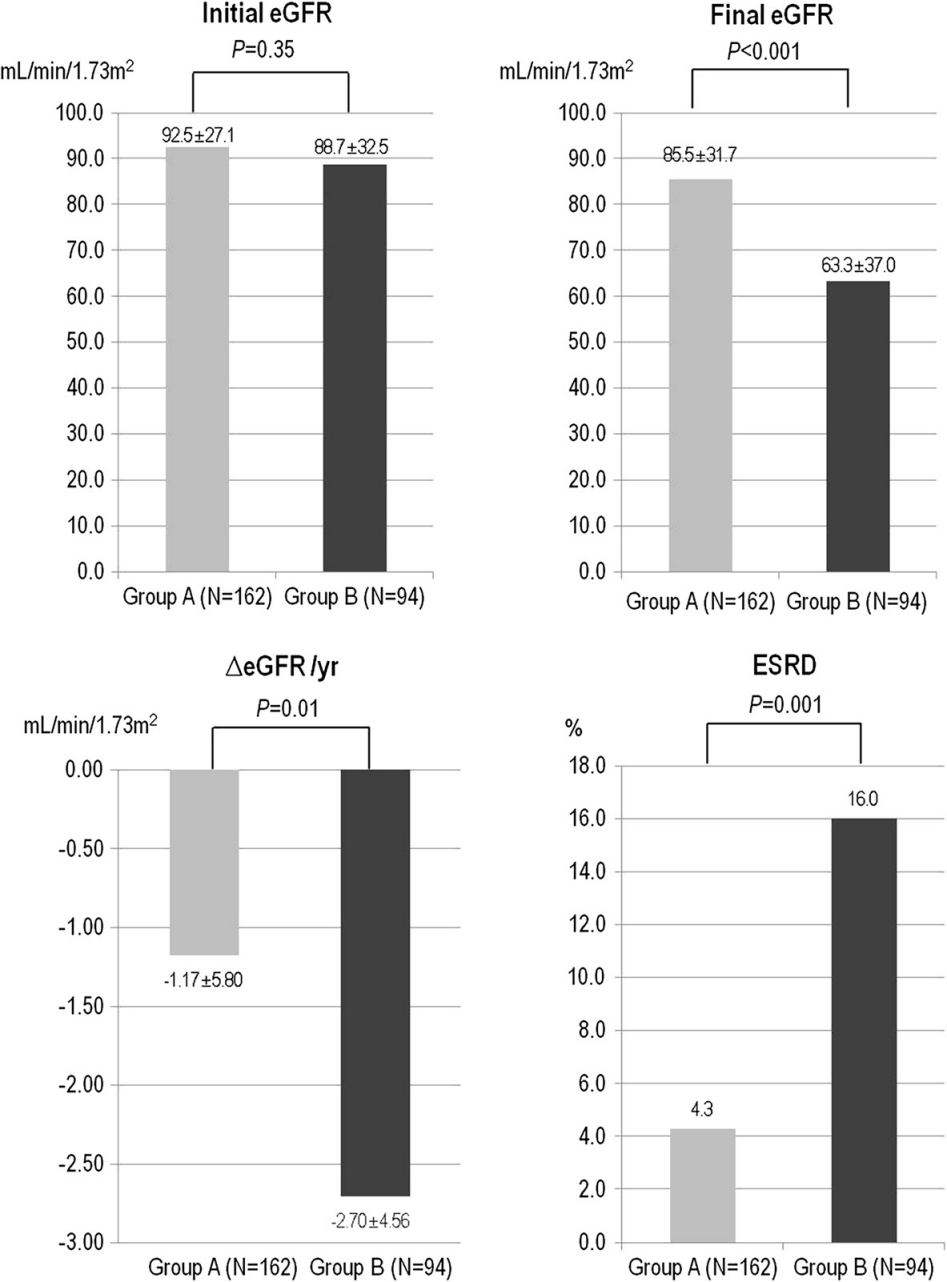
**Renal outcomes of patients according to asymptomatic pyuria.** There was no significant difference between group A and group B in initial eGFR (92.5 ± 27.1 vs. 88.7 ± 32.5 ml/min/1.73m^2^; P=0.35). However, the final eGFR was significantly lower in group B than in group A (63.3 ± 37.0 vs. 85.5 ± 31.7 ml/min/1.73m^2^; P<0.001). Moreover, ΔeGFR was larger in group B than in group A (-2.7 ± 4.56 vs. -1.17 ± 5.8 ml/min/1.73m^2^ per year, respectively; P=0.01). The incidence of ESRD was also significantly higher in group B than in group A (n = 15, 16.0% vs. n=7, 4.3%; P=0.001). eGFR, estimated glomerular filtration rate; ESRD, end-stage renal disease.

The initial eGFR at the first office visit did not differ between group A and group B. However, the final eGFR in group B was significantly lower than that in group A (63.3 ± 37.0 vs. 85.5 ± 31.7 ml/min/1.73m^2^, respectively; *P* < 0.001). Because the follow-up duration can affect the final eGFR value, we compared the annual change in eGFR (ΔeGFR) between group A and group B and found that group B showed a faster decline in eGFR than group A (-2.7 ± 4.56 vs. -1.17 ± 5.8 ml/min/1.73m^2^ per year, respectively; *P* = 0.01; Figure 2). When we performed a subgroup analysis in baseline CKD stage I-II, group B showed even greater annual decline of eGFR compared to group A in CKD (-3.36 ± 3.47 vs. -1 ± 5.97 ml/min/1.73m^2^ per year, *P* = 0.002, Additional file 2: Figure S2). However, when we performed the independent *t*-test in the prospective groups based on the number of pyuria episodes in the first year (Group^no pyuria/1st year^ and Group^1-4 pyuria/1st year^), the difference in GFR decline rate disappeared (Additional file 3: Table S1 and Additional file 4: Table S2).

To analyze the risk factors that are associated with ΔeGFR, we performed a linear regression analysis. In a univariate analysis, age, diabetes mellitus, hypertension, overt UTI, and group B were associated with ΔeGFR. In a multiple linear regression model, group B was revealed as an independent risk factor for a faster decline in eGFR (B = -1.53, 95% confidence interval -2.904 to -0.155; *P* = 0.03; Table 6). More patients in group B (n = 15, 16.0%) reached ESRD during observational period compared to the patients in group A (n = 7, 4.3%; *P* = 0.001; Figure 2).

**Table 6:** Independent factors associated with the annual change of ΔGFR

**Variable**	B*	**95% CI for *B***		**P-value**
		**Lower**	**Upper**	
Age	-0.081	-0.133	-0.030	0.002
HTN (initial)	-2.394	-3.767	-1.021	0.001
DM (initial)	-6.076	-12.246	0.095	NS
Group B^†^	-1.530	-2.904	-0.155	0.03
Overt UTI^†^	-2.162	-4.140	-0.184	0.03

Analyzed by multiple linear regression model, *R*^*2*^ = 0.101 for 3 covariates with Group B, *R*^*2*^ = 0.093 for 3 covariates with Overt UTI. The age, initial HTN and initial DM were analyzed as covariates with Group B or Overt UTI.

*B: Unstandardized coefficients.

^†^ Age, initial HTN and initial DM were analyzed as covariates with Group B or Overt UTI.

ΔGFR, annual reduction rate of glomerular filtration rate; CI, confidence interval; DM, diabetes mellitus; HTN, hypertension; UTI, urinary tract infection.

### Annual eGFR Reduction Rate in the ADPKD Patients with Chronic Pyuria

To control for the possible confounding effects of overt UTI in group B, we excluded patients who experienced overt UTI from both group A and group B. The subsequent cohorts were renamed group A^UTI-^ and group B^UTI-^_,_ respectively. The patients who experienced overt UTI were separately classified as the overt UTI group irrespective of whether they were in group A or group B.

**Figure 3:**
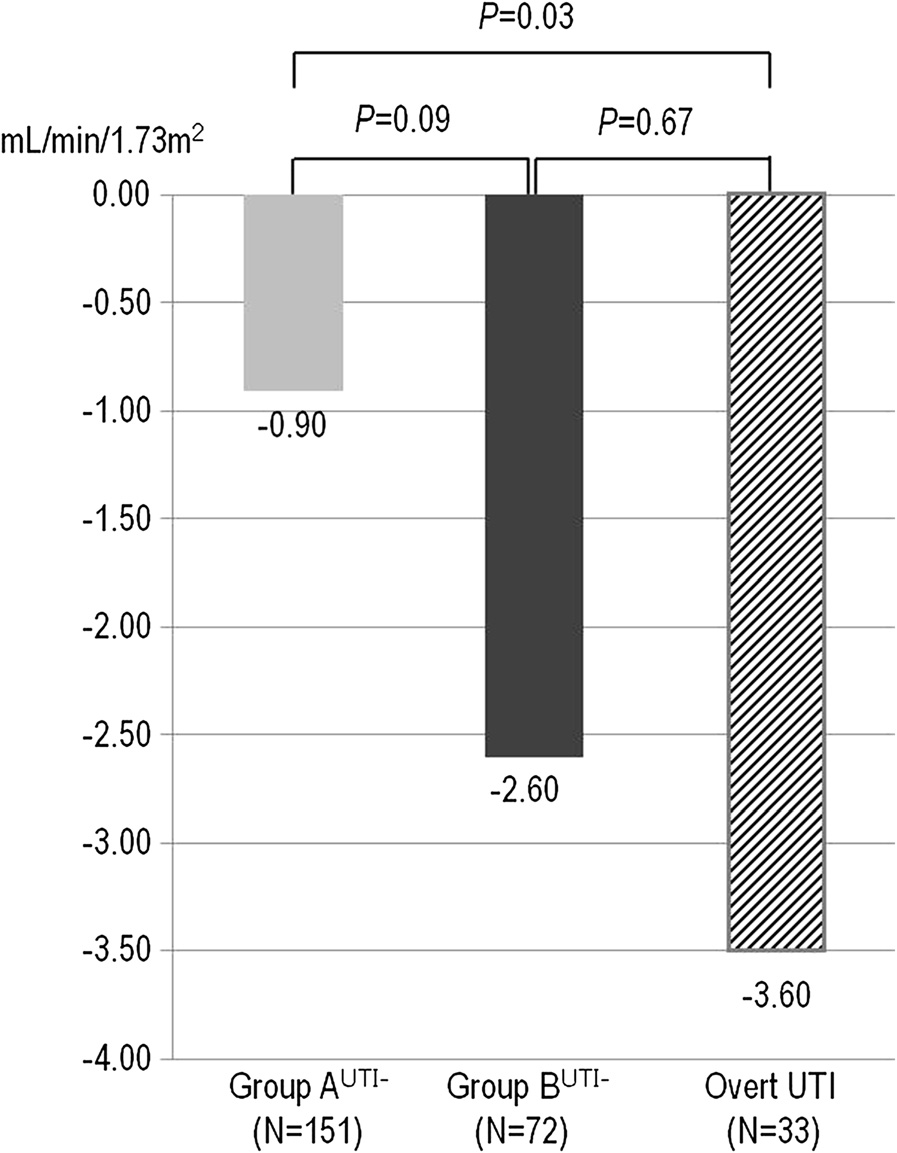
**Comparison of ΔeGFR according to the occurrence of overt UTI and pyuria type.** To Evaluate the effects of asymptomatic pyuria and overt UTI on ΔeGFR, the incidence of UTI and the pyuria type divided into 3 groups. Group A^UTI-^, non-pyuria or transient pyuria without an overt UTI episode; group B^UTI-^, recurrent or persistent pyuria without overt UTI; overt UTI, at least one episode of UTI. UTI, urinary tract infection.

A total of 151 patients in group A^UTI-^, 72 patients in group B^UTI-^, and 33 patients in the overt UTI group were included in the analysis. The group B^UTI-^ patients exhibited a greater ΔeGFR compared to group A^UTI-^ (-2.6 vs. -0.9 ml/min/1.73m^2^ per year, respectively; *P =* 0.009; Figure 3). The overt UTI group patients exhibited an even greater ΔeGFR at 1 year after their UTI episode (-3.6 ml/min/1.73m^2^ per year). The clinical characteristics, including ΔeGFR, were compared between the groups (Table 7). There were statistically significant differences in gender, follow-up duration, final eGFR, ΔeGFR, and the incidence of ESRD between group A^UTI-^ and other groups. However, the differences between group B^UTI-^ and the overt UTI group were not statistically significant.

**Table 7:** Clinical characteristics according to the occurrence of UTI and pyuria type

		**No UTI (N=223)**		**Overt UTI (N=33)**	***P *value**^**†**^
		**Group A**^**UTI-**^**(N=151)**	**Group B**^**UTI-**^**(N=72)**		
Age^*^		47.8 ±13.5	47.2 ± 11.3	51.2 ± 11.8	NS
Female	number (%)	63 (41.7)	39 (54.2)	21 (63.6)	0.04
F/U Duration	Months	51.0 ± 40.3	86.2 ± 37.5	83.1 ± 43.3	<0.001
HTN (initial)	number (%)	90 (59.6)	51 (70.8)	26 (78.8)	NS
Stone (initial)	number (%)	22 (14.6)	12 (16.7)	6 (18.2)	NS
Initial eGFR^‡^	ml/min/1.73m^2^	93.4 ± 26.7	89.2 ± 33.0	84.7 ± 30.8	NS
Final eGFR^‡^	ml/min/1.73m^2^	87.6 ± 30.1	64.9 ± 38.1	57.6 ± 35.2	<0.001
ΔeGFR^‡^	ml/min/1.73m^2^/year	-0.9 ± 5.7	-2.6 ± 4.4	-3.6 ± 5.6	0.009
ESRD	number (%)	5 (3.3)	11 (15.3)	6 (18.2)	0.001

Group A^**UTI-**^ and B^**UTI-**^ were classified as patients in group A and group B without overt UTI. *mean ± standard deviation (SD).

† *P* value calculated using the One-Way ANOVA. Post-hoc analyses were performed using the Scheffe’s method.

^‡^ Estimated glomerular filtration rate (eGFR) was calculated using the CKD-EPI equation (ml/min/1.73m^2^).

ΔeGFR, annual reduction rate of estimated glomerular filtration rate; eGFR, estimated glomerular filtration rate; ESRD, end stage renal disease; F/U, follow-up; HTN, hypertension; UTI, urinary tract infection.

## Discussion

Although asymptomatic pyuria is quite common in patients with ADPKD, its etiology, incidence, and clinical implications are poorly documented. We observed that chronic asymptomatic pyuria (group B) was associated with an increased incidence of overt UTI and ESRD and with greater ΔeGFR. In addition, group B was an independent risk factor for ΔeGFR when adjusted for age, follow-up duration, and comorbidity.

In our study, chronic asymptomatic pyuria was associated with overt UTI. The group B showed higher occurrence rate of overt UTI and upper UTI. This was true when we performed the analysis in the prospective cohort based on the number of pyuria episodes in the first year of follow up. The patients who have experienced pyuria in the first year showed higher incidence of overt and upper UTI in the following periods. Although urine culture was performed in very few cases, our study result suggests that asymptomatic pyuria is the risk factor for subsequent UTI events and therefore ADPKD patients with pyuria should be closely monitored in the outpatient clinic. In our study, a total of 50 episodes of overt UTI occurred in 33 (12.9%) patients over approximately 5 years. The incidence of overt UTI (0.014 episodes/patient/year) in our patients was similar to the incidence that was reported recently by Sallee et al. (0.011 episodes/patient/year) [13].

Chronic asymptomatic pyuria and overt UTI were also associated with ΔeGFR in the ADPKD patients. The patients in group B exhibited a greater ΔeGFR (-2.7 ± 4.56 vs. -1.17 ± 5.8 ml/min/1.73m^2^ per year; *P* = 0.01) and a higher incidence of ESRD (16.0% vs. 4.3%, *P* = 0.001) than the patients in group A. There have been some reports about the proportion of asymptomatic pyuria in normal population. However, it has not been studied how chronic asymptomatic pyuria affects renal functions [18,19]. The finding that the microorganism grown in the urine cultures of asymptomatic pyuria patients was similar to the common pathogen found in urinary tract infections may suggest that asymptomatic pyuria may be a type of subclinical bacterial infection. Because chronic infection is a risk factor for renal function deterioration, chronic asymptomatic pyuria may be a form of undetected subclinical bacterial infection that causes progressive renal function deterioration.

Another explanation may be that chronic asymptomatic pyuria or overt UTI is a direct cause of cystogenesis. Many hypotheses have been proposed for the generation and progression of renal cysts in ADPKD patients. The two-hit model that was proposed by Germino et al. suggested that a somatic mutation on the opposite allele might lead to a loss of heterozygosity [20-24]. In their theory, the 1^st^ hit refers to the germline mutation, and the 2^nd^ hit refers to the somatic mutation. However, the 2^nd^ hit does not always lead to an instant renal cyst formation [25-27]. The partial loss of the protein polycystin in primary cilia did not induce a change in adult renal tubular epithelial cells. However, when a 3^rd^ hit such as ischemic damage [25,27] or nephrotoxic injury [26] was applied to the renal tubular epithelial cells, abnormal cellular proliferation and the cyst formation were observed [10,28,29]. A previous study of germ-free Sprague–Dawley rats found that endotoxins and nordihydroguaiaretic acid can induce renal cystic disease and leukocyte-mediated renal damage independent of a secondary infection [30]. In addition, the roles of TNF-alpha in cyst formation [31] and lipopolysaccharide (LPS) in renal deterioration by peritubular capillary dysfunction [32] have been reported. Therefore, inflammation caused by overt UTI or chronic asymptomatic pyuria itself can serve as the 3^rd^ hit and may lead to the cellular proliferation, cyst formation and fibrosis. However, there is still a possibility that asymptomatic pyuria is merely a marker of renal progression rather than a risk factor for future GFR decline. In order to elucidate causal relationship between asymptomatic pyuria and GFR decline, a well-designed prospective study is warranted.

The limitation of this study is that it was a single-center, retrospective study. Because the number of episodes of overt UTI was low, we could not detect a statistically significant difference in ΔeGFR between the chronic pyuria and overt UTI groups. Among the overt UTI episodes, 7 out of 20 cases were not associated with a loss of eGFR. Therefore, further prospective studies are needed to determine how chronic asymptomatic pyuria and overt UTI may cause a loss of eGFR. Moreover, we did not measure either cyst volume or kidney volume as an outcome. However, measuring total kidney volume should be considered in the future studies, as this parameter has been used as a surrogate marker for renal function deterioration in the early stage of the disease [33,34]. Finally, urine culture study was only performed in very few cases of pyuria. Whether asymptomatic pyuria is a form of inflammation or asymptomatic bacteriuria should be documented in further studies.

The present study shows that chronic asymptomatic pyuria increases the incidence of overt UTI and may contribute to declining renal function in ADPKD patients. To the best of our knowledge, this is the first report that chronic asymptomatic pyuria may precede overt UTI and renal function deterioration in ADPKD patients. Our results support a previous report by Idrizi et al., which suggested that chronic treatment with antibiotics can preserve renal function. Although the mechanism of renal function deterioration with chronic pyuria is unclear, it is worthwhile to attempt to control chronic pyuria in ADPKD patients.

## Conclusions

In conclusion, we found that chronic asymptomatic pyuria may increase the risk of developing overt UTI and may contribute to declining renal function in ADPKD. Further prospective study is warranted to reveal the causal relationship between chronic pyuria and the development of overt UTI and renal function deterioration.

## Abbreviations

ADPKD: Autosomal dominant polycystic kidney disease; ANOVA: Analysis of variance; APN: Acute pyelonephritis; CKD-EPI: Chronic Kidney Disease Epidemiology; ΔeGFR: Annual change in estimated glomerular filtration rate; eGFR: Estimated glomerular filtration rate; ESRD: End-stage renal disease; Group A: No pyuria or transient pyuria group; Group A ^UTI-^: Group A without overt UTI; Group B: Chronic pyuria group (recurrent pyuria or persistent pyuria group); Group B ^UTI-^: Group B without overt UTI; Group^no pyuria/1st year^: Group who did not experience any pyuria episodes in the first year; Group^1-4 pyuria/1st year^: Group who experienced 1-4 pyuria epidoses during the first year; HPF: High-power field; LPS: Lipopolysaccharide; sCr: Serum creatinine; UTI: Urinary tract infection; WBC: White blood cell.

## Competing interests

This work was supported by a grant (A080588) from the Korea Healthcare Technology R&D Project, Ministry for Health, Welfare and Family Affairs, Republic of Korea. This paper has not been published previously in whole or part, except in abstract format. Curie Ahn: C Ahn received the grant (A080588) from the Korea Healthcare Technology R&D Project, Ministry for Health, Welfare and Family Affairs, Republic of Korea (2009~2012).

## Authors’ contributions

JH and HP participated in the design of the study and drafted the manuscript. JJ, SY, and KO helped to revise the manuscript. SB and MH helped to collect the clinical data. KB and JY participated in the statistical analyses. JC carried out imaging analysis to give a help in differential diagnosis of APN and cyst infection. HP, YH, CA enrolled the patients at the clinic and collected sample and clinical information. CA conceived of the study, and participated in its design and coordination, interpreted the data, and helped to draft and revise the manuscript. All authors read and approved the final manuscript.

## Pre-publication history

The pre-publication history for this paper can be accessed here:

http://www.biomedcentral.com/1471-2369/14/1/prepub

## Supplementary Material

Additional file 1 **Figure S1.** Higher incidence of overt urinary tract infection (UTI) and upper UTI in the pyuria group in the first year. The group of patients with 1–4 pyuria episodes in the first year of follow up (Group^1-4pyuria/1st year^) had higher incidence of overt UTI and upper UTI compared to the no pyuria group (Group^no pyuria/1st year^). Click here for file

Additional file 2 **Figure S2.** Chronic pyuria group (Group B) shows greater annual decline of eGFR in the conserved renal function group (chronic kidney disease stage I-II). In CKD I-II group, chronic pyuria group (group B) showed much greater annual decline of eGFR compared to group A (−3.36 ± 3.47 vs. -1 ± 5.97 ml/min/1.73m^2^ per year, *P* = 0.002). The follow up duration between the groups were significantly different (Group A vs. Group B, 54.12 ± 41.0 vs. 95.08 ± 34.3 months). Click here for file

Additional file 3 **Table S1.** Frequency of pyuria in the 1^st^ year of follow up. Among 256 patients, 129 patients did not experienced any pyuria episode in the 1^st^ year of follow up. The other 127 patients experienced ≥1 episode of pyuria in the initial year. Click here for file

Additional file 4 **Table S2.** Greater annual eGFR decline in the initial pyuria group (Group^1-4pyuria/1st year^) compared to the no pyuria group (Group^no pyuria/1st year^). Although statistically insignificant, the initial pyuria group (Group^1-4pyuria/1st year^) showed greater annual eGFR decline rate compared to the no pyuria group (Group^no pyuria/1st year^) (−2.25 ± 6.38 vs. -1.21 ± 4.24 mL/min/1.73m^2^/year, *P* = 0.127). Click here for file
