# Supplementary figures and images for: Chronic asymptomatic pyuria precedes overt urinary tract infection and deterioration of renal function in autosomal dominant polycystic kidney disease
Source: BMC Nephrol. 2013 Jan 7;14:1. doi: 10.1186/1471-2369-14-1 (PMC3545884; doi:10.1186/1471-2369-14-1)

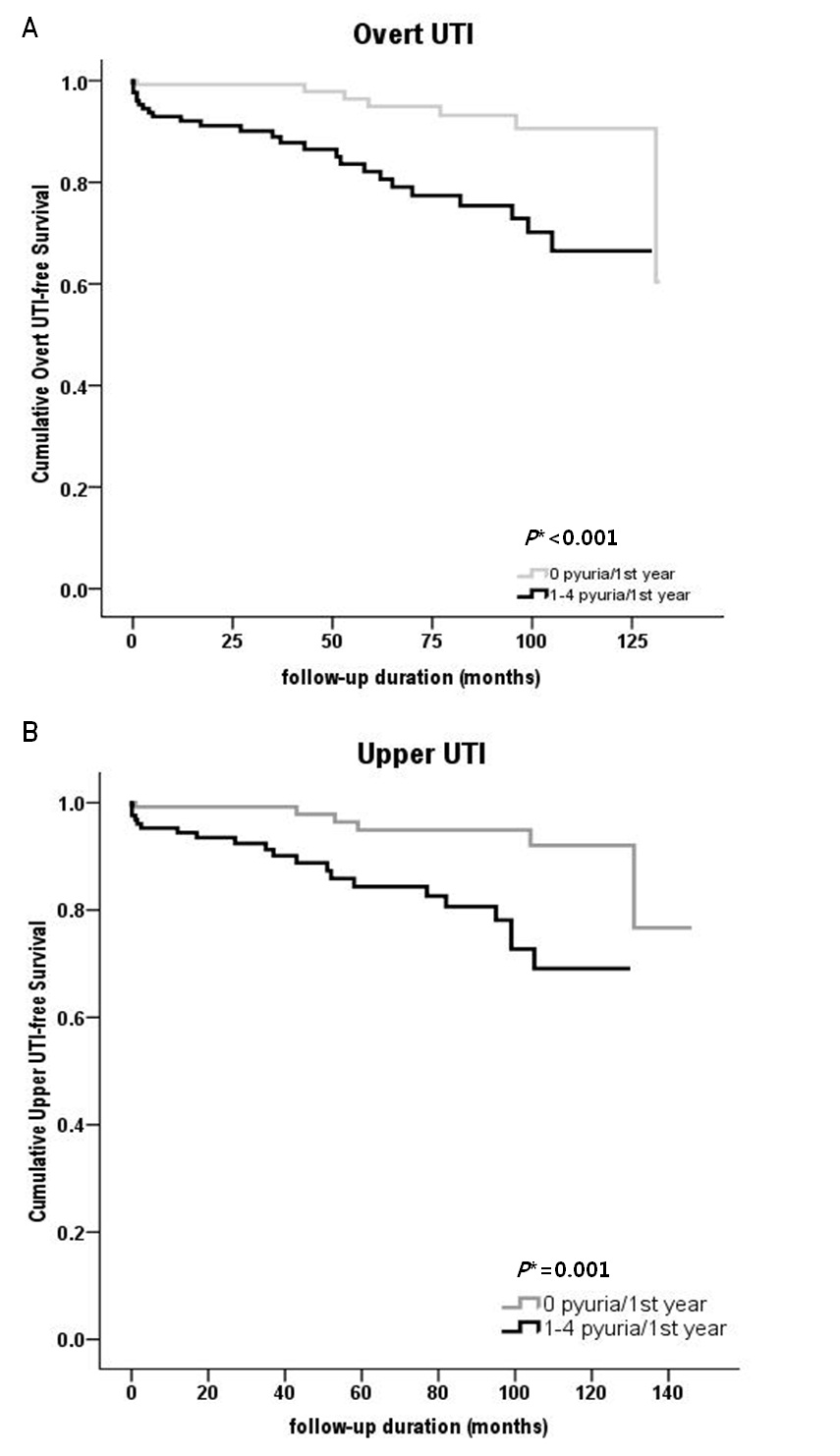

Supplement: Additional file 1 — Figure S1. Higher incidence of overt urinary tract infection (UTI) and upper UTI in the pyuria group in the first year. The group of patients with 1–4 pyuria episodes in the first year of follow up (Group1-4pyuria/1st year) had higher incidence of overt UTI and upper UTI compared to the no pyuria group (Groupno pyuria/1st year). [file 1471-2369-14-1-S1.jpeg]

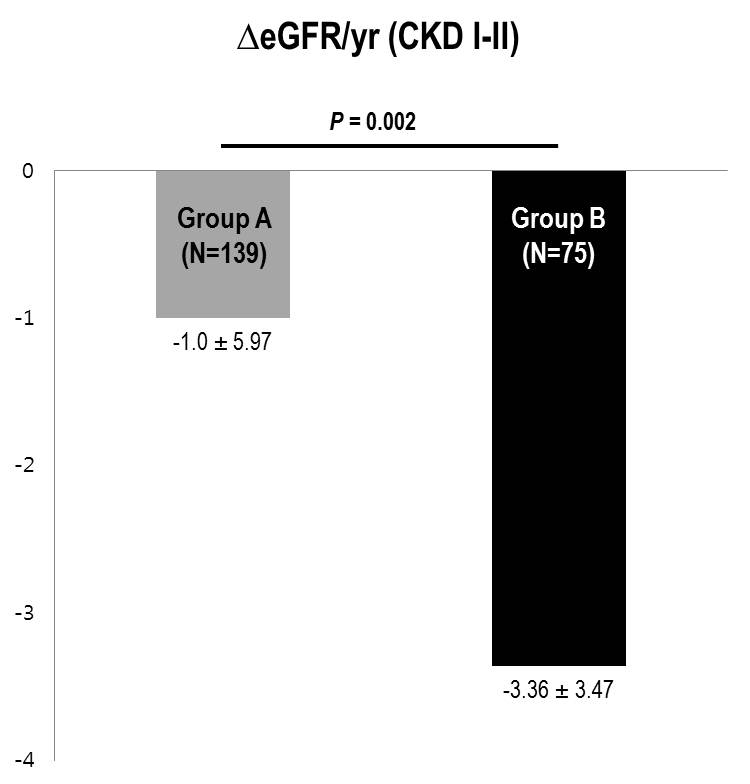

Supplement: Additional file 2 — Figure S2. Chronic pyuria group (Group B) shows greater annual decline of eGFR in the conserved renal function group (chronic kidney disease stage I-II). In CKD I-II group, chronic pyuria group (group B) showed much greater annual decline of eGFR compared to group A (−3.36 ± 3.47 vs. -1 ± 5.97 ml/min/1.73m2 per year, P = 0.002). The follow up duration between the groups were significantly different (Group A vs. Group B, 54.12 ± 41.0 vs. 95.08 ± 34.3 months). [file 1471-2369-14-1-S2.jpeg]

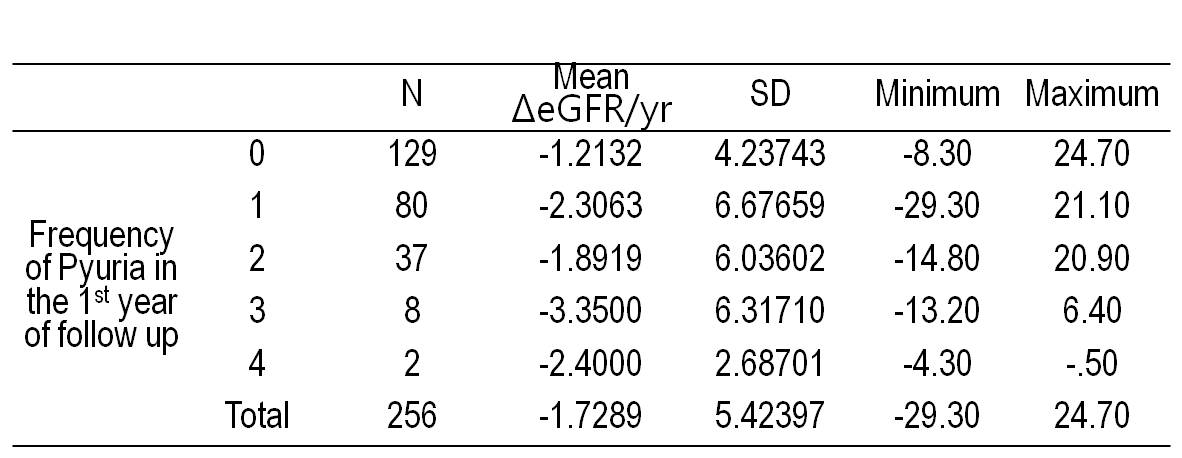

Supplement: Additional file 3 — Table S1. Frequency of pyuria in the 1st year of follow up. Among 256 patients, 129 patients did not experienced any pyuria episode in the 1st year of follow up. The other 127 patients experienced ≥1 episode of pyuria in the initial year. [file 1471-2369-14-1-S3.jpeg]

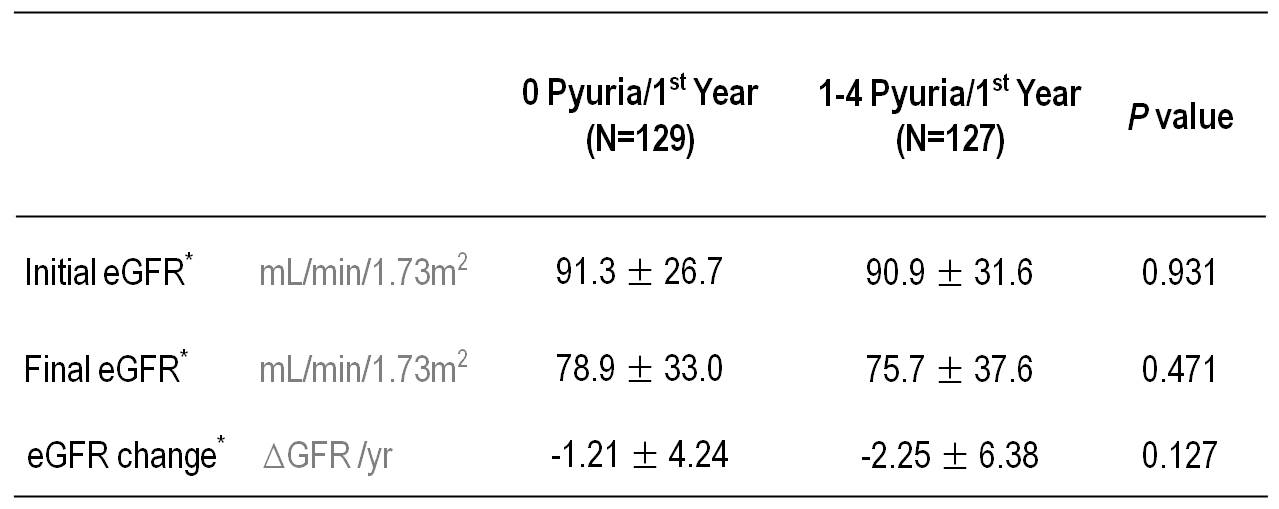

Supplement: Additional file 4 — Table S2. Greater annual eGFR decline in the initial pyuria group (Group1-4pyuria/1st year) compared to the no pyuria group (Groupno pyuria/1st year). Although statistically insignificant, the initial pyuria group (Group1-4pyuria/1st year) showed greater annual eGFR decline rate compared to the no pyuria group (Groupno pyuria/1st year) (−2.25 ± 6.38 vs. -1.21 ± 4.24 mL/min/1.73m2/year, P = 0.127). [file 1471-2369-14-1-S4.jpeg]
